# Qualitative Comparative Analysis of exercise interventions for fall prevention in residential aged care facilities

**DOI:** 10.1186/s12877-024-05246-0

**Published:** 2024-09-03

**Authors:** Jenni Suen, Rik Dawson, Dylan Kneale, Wing Kwok, Catherine Sherrington, Katy Sutcliffe, Ian D. Cameron, Suzanne M. Dyer

**Affiliations:** 1 Rehabilitation, Aged and Extended Care, Flinders Health and Medical Research Institute, College of Medicine and Public Health, Flinders University of South Australia, Bedford Park, Australia; 2 Institute for Musculoskeletal Health, The University of Sydney and Sydney Local Health District, Sydney, NSW Australia; 3 EPPI Centre, UCL Social Research Institute, University College London, 27 Woburn Square, London, WC1H 0NS UK; 4 John Walsh Centre for Rehabilitation Research, Northern Sydney Local Health District and the University of Sydney, St Leonards, Australia; 5 Rehabilitation, Aged and Extended Care Flinders Medical Centre, GPO Box 2100, Adelaide, SA 5001 Australia

**Keywords:** Fall prevention, Exercise intensity, Care home, Nursing home, Exercise program

## Abstract

**Background:**

Exercise interventions are highly effective at preventing falls in older people living in the community. In residential aged care facilities (RACFs), however, the evidence for effectiveness is highly variable, warranting exploration of drivers of successful trials. This study aims to identify the conditions of randomised controlled trials (RCTs) that are associated with reducing falls in RACFs and test whether it can explain the variability.

**Methods:**

RCTs testing exercise interventions in RACFs compared to usual care, reporting rate or risk of falls from the 2018 Cochrane Collaboration review and a search update to December 2022 were included. Two authors independently extracted and coded trial conditions and outcomes according to a theory developed from prior Intervention Component Analysis. Trial outcomes were coded as successful or unsuccessful based on point estimates for the rate or risk ratio for falls, or *p* value. Qualitative Comparative Analysis (QCA), utilising Boolean minimisation theory, was conducted to determine the key conditions driving trial success. A subgroup meta-analysis and the GRADE approach was applied to the final theory.

**Results:**

Eighteen trials undertaken in 11 countries with 2,287 residents were included. Participants were predominately ambulant females aged 70 to 80 with cognitive impairment. Most interventions were fully supervised or supervised at the start of the intervention. QCA identified two configurations as drivers of successful exercise falls prevention programs: (i) group exercise that is moderate or low intensity, or (ii) for independent ambulatory residents, exercise for more than 1 h per week. The combination of configuration (i) and (ii) had consistency and total coverage scores of 1, indicating all trials were explained. This combination was associated with a reduction in falls (rate ratio 0.45, 95%CI 0.34 to 0.59; risk ratio 0.66, 95%CI 0.53 to 0.82; low certainty evidence).

**Conclusion:**

To successfully reduce falls in RACFs, exercise programs should provide continuous supervised moderate-intensity group exercise. For programs that mostly include independent ambulatory residents, exercise for at least 80 min per week should be provided. As many current residents are frail, tailored exercise is likely necessary and an individualised dose may be required. Future trials should test exercise interventions for less mobile residents.

**Supplementary Information:**

The online version contains supplementary material available at 10.1186/s12877-024-05246-0.

## Introduction

Exercise interventions can facilitate safe mobility by enhancing muscle strength and balance and the ability to avoid falls [1, 2]. Thus, exercise is a featured and recognised intervention approach in falls prevention guidelines [3, 4]. In the community, there is high certainty evidence that balance and functional exercise interventions for falls prevention can lead to a 24% reduction in the rate of falls amongst older adults [2]. However, in residential aged care facilities (RACFs), the evidence is highly variable, leading to a conclusion that the effect of exercise on the rate of falls was not certain in the most recent 2018 Cochrane Collaboration review [5].

RACFs are a complex environment in which to provide exercise interventions. Exercise professionals are required to integrate with the organisation through varied funding schemes and work with a high turnover of nursing staff [6, 7]. Residents are a vulnerable group of older adults with a high falls risk due to falls history, cognitive impairment, frailty, and malnutrition [8, 9]. Despite these environmental complexities, a recent reanalysis and update of exercise intervention trials in RACFs demonstrated a beneficial effect on falls prevention [10]. There was moderate certainty evidence that exercise interventions in RACFs reduce falls at the end of the exercise program, however there was no lasting effect on falls reduction observed after the exercise program had ended (high certainty evidence) [10]. Continuing exercise programs in RACFs is therefore required to prevent falls amongst residents [10]. However, the considerable heterogeneity observed between trials (I^2^ = 85%), where some trials clearly reduced falls and others increased falls, warrants further exploration to understand the differences between successful and unsuccessful exercise interventions for falls prevention [11].

A recent Intervention Component Analysis (ICA) identified a theory of possible drivers of successful falls prevention exercise interventions from the perspective of the trialists [12, 13]. The theory stated that the provision of the ‘right’ exercise with ‘sufficient resourcing’ may be required for a falls prevention exercise intervention to reduce falls in RACFs [14]. Examining this ICA theory further through Qualitative Comparative Analysis (QCA) can test and refine the theory to determine the best combination of trial conditions that explains the success and failure of the existing trials to prevent falls [14].

QCA has been recently applied in systematic reviews because of its suitability for examining intervention complexity [15–17]. Based on an assumption that complex interventions differ in multifarious ways and that outcomes result from the interplay of an intervention with its implementation and context, QCA combines processes and principles of qualitative inquiry with quantitative analytical methods [14]. This approach identifies critical conditions related to the intervention and/or contextual elements [14]. The underlying logic of QCA, derived from set theory, involves exploring how sets of trials with similar characteristics overlap with sets of trials defined as having a successful or unsuccessful outcome. As a result, this approach can identify conditions associated with successful trials with complex interventions.

This study had two aims. The first aim was to use QCA to test the components identified from ICA as an initial theory (i.e., combination of right exercise and sufficient resourcing) [13] to examine if this theory explains the success and failure of all included trials at preventing falls in RACFs and to refine the theory as required. The second aim was to determine if the combination of exercise trial conditions identified in QCA could explain the heterogeneity within the meta-analyses of rate or risk of falls from trials examining exercise for falls prevention in RACFs [5, 10].

## Methods

This study is a QCA, a synthesis method of qualitative information in systematic reviews [18]. This QCA follows on from an ICA [13] which identified a theory and theory components which can be tested in QCA to determine the critical conditions important for trials to be successful [14]. This QCA also specifically follows on from the trial endpoint meta-analysis of exercise interventions associated with an update of the 2018 Cochrane Collaboration meta-analysis where the end of intervention meta-analysis demonstrated an overall reduction in falls, but considerable heterogeneity amongst trials remained [10].

### Search strategy and selection process

Trials included in the 2018 Cochrane Collaboration review [3] plus records from an additional search update of CENTRAL, MEDLINE, Embase and CINAHL Plus databases from 2017 to December 2022 were screened to identify eligible trials [5], consistent with the ICA and endpoint meta-analysis [10, 13]. Trial records and conference abstracts were not systematically searched or included. Records retrieved from the updated search were imported into Endnote × 9 (Clarivate Analytics, PA, USA) for duplicate removal. The remaining records were imported into Covidence (Veritas Health Innovation, Melbourne Australia) for independent screening by two authors. Any discrepancies were discussed with a third author as necessary. Based on the recent trial endpoint meta-analysis, only trials with end of intervention data were included [10].

#### Inclusion criteria

Randomised control trials (RCTs) that tested exercise interventions compared to usual care in RACFs, reported data suitable for calculating the ratio of the rate or risk of falls at the end of the intervention, or reporting falls data with a *p*-value when effect estimates and confidence intervals were not available, were included.

#### Top up search

A top up search from December 2022 to May 2024 was conducted consistent with the search strategy described above, restricted to RACFs (Appendix 1). Two reviewers independently screened the records to identify any additional trials published subsequent to those included in the QCA. Eligible studies from this search, their characteristics, outcomes, and alignment with the final QCA theory were discussed. Updating the QCA analysis was not undertaken as the findings of an analysis of this type are considered at a low risk of being outdated after 12 months [19]. Instead, any additional published studies are likely to either provide further support for the final theory or add nuances to the original findings, rather than alter the final conclusion [19].

### Data extraction and risk of bias

Two authors independently extracted study characteristics (i.e. number randomised, mean age, percent female, intervention and control description) and end of intervention falls outcomes data (i.e., rate ratio or risk ratio or data enabling calculation of these outcomes) from new trials identified from the search update using a proforma or in Covidence [5]. A study characteristics table was generated.

To form a Data Table for QCA; intervention, implementation, participant and trial design characteristics identified by the ICA were extracted [13]. The ICA theory indicated that ‘right exercise’ comprising a targeted balance and strength program tailored to the individual, along with the delivery of moderate-intensity exercise and sufficient resources to deliver structured and supervised exercise at an adequate dose, was important for falls prevention exercise programs to be successful in RACFs [13]. Thus, intervention characteristics of progressive standing strength and balance, tailored, moderate intensity, right exercise and implementation characteristics of sufficient resourcing, group exercise, falls education, and supporting exercise engagement were extracted for the Data Table using information from all published records associated with included trials (consistent with Dawson and colleagues supplementary materials [13]), based on the methods of Sutcliffe and colleagues [14]. The main themes of the theory were right exercise and supporting exercise engagement, with subthemes of balance and strength, tailored, moderate intensity, sufficient resourcing, group exercise and falls education [13]. As described in the ICA, low-intensity exercise refers to gentle physical activity where the heart rate and breathing are low and moderate intensity exercise was defined as exercise that elevates heart rate and breathing moderately, as judged by physiotherapist authors based on the Borg scale of perceived exertion [13, 20]. Based on trialists’ views, conditions including tailored, plus participant characteristics of cognitive impairment and mobility were added to the Data Table [13]. Trial design characteristics of study quality and small sample size were also extracted [13]. Study quality was based on the Physiotherapy Evidence Database (PEDro) scale [13, 21].

For trial outcomes, ratios for the rate and risk of falls between trial arms, at the end of the intervention period were extracted from Dyer and colleagues [10] and the 2018 Cochrane Collaboration review [5]. Ratios for rate of falls (i.e., the total number of falls per unit of person time that falls were monitored during the intervention) was used in preference to risk of falls (i.e., the number of fallers during the intervention), when reported, as this outcome appears more sensitive to change [2, 5].

### Qualitative comparative analysis

#### Data table

Trial conditions identified for data extraction were coded independently by two physiotherapist authors as intervention conditions absent (0), present (1), or by degree of presence (numbers from 0 to 1 excluding 0.5). Successful trials were coded as 1 for statistically or clinically significant at reducing falls (point estimate ≤ 0.8 or *p* < 0.05 where point estimates were not available). Unsuccessful trials were coded as 0 for no effect on falls (point estimate > 0.8 to < 1.2) or increased falls (point estimate ≥ 1.2). Any discrepancies between coding of conditions were resolved by discussion between two authors, involving a third author as necessary.

#### Analysis

QCA was conducted in R Project (version 4.3.1, 2023–06-16, 64bit) using the graphical user interface (GUI) [22]. The Data Table of coded intervention conditions (analogous to factors or variables) and outcomes was imported into the GUI. The intervention components of the theory from ICA were included in a Truth Table to determine the degree of inclusion of each component in impacting trial outcomes. In a Truth Table, each row represents a single or multiple studies with the same configuration of characteristics [22]. The results of the first Truth Table determined if a modified theory should be tested in a refined Truth Table or if the initial theory should progress to the next step of QCA.

The ICA theorised that (a) “right exercise” by targeting balance and strength, tailored to the individual and delivered simply at a moderate intensity and (b) “sufficient resourcing” to deliver structured and supervised exercise at an adequate dose were both important in driving trial success from the perspective of trialists. These two conditions were first tested in a Truth Table accordingly [13]. Subsequent ‘theories’ were developed for exploration that represented adaptations to the original theory.

**Fig. 1:**
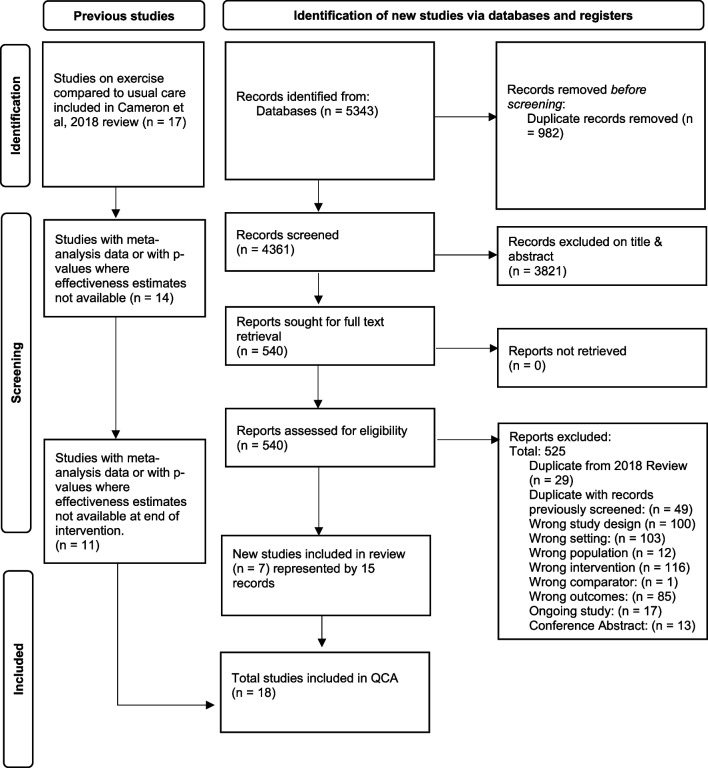
Qualitative comparative analysis process

For each theory, in line with QCA guidance [17, 18, 22, 23], the following QCA steps were conducted, summarised in Fig. 1:

1. *Truth Table analysis.* The conditions of the theory (i.e., right exercise and sufficient resourcing) were tested in this analysis to determine if they differentiated successful trials from unsuccessful trials.

QCA is based on set theory which seeks to determine whether conditions are either necessary (i.e. the intended outcome will only occur in the presence of those conditions) or sufficient (i.e. the conditions are enough to generate the intended outcome, but alternative conditions might also generate the outcome). Our analysis sought to identify a relationship of sufficiency.

The Truth Tables include two scores (i.e., inclusion score and consistency score) that illustrate the strength of the relationship between the configuration and a successful outcome by demonstrating how many successful and unsuccessful trials were explained by the Boolean groups (i.e., configurations) of the conditions in the theory.

The inclusion score, or sufficiency inclusion score, indicates the proportion of trials represented by the configuration and the extent to which the combination of conditions can be considered sufficient to trigger falls prevention. Sufficiency in this case indicates the extent to which we can consider a set of trials with a particular set of characteristics as also being a subset of the set of successful trials. A score of zero indicates that no trials with that configuration of conditions were in the set of trials successful at preventing falls. A score of 1 indicates that all trials with that configuration of conditions successfully prevented falls, suggesting that this condition or configuration of conditions is ‘sufficient’ for success [24]. Scores between zero and 1 indicate various degrees of consistency, where a score of 0.5 represents maximum ambiguity as to whether a sufficient relationship exists.

Proportion Reduction in Inconsistency (PRI) scores are an additional measure used to assess the strength of the relationship between the configuration and outcome, but also incorporate the extent to which configurations triggering falls prevention are not simultaneously triggering the opposite outcome (where a score of 1 or close to 1 indicates a sufficient relationship).

If the configurations of conditions tested in the Truth Table analysis were not able to explain the success or failure of most or all included trials, the inclusion and consistency scores were used to identify which conditions provided a better explanation for the trial outcomes (i.e. scores closer to 1) to retain for further Truth Table analyses. Successful trials not explained by the configuration of conditions tested are also identified. Additional conditions related to unexplained successful trials were identified from the Data Table and considered for examination in further Truth Table analyses.

2) *Minimisation:* If the configurations had inclusion and consistency scores which explained the success or failure of most or all included trials (i.e. scores close to 1), Boolean minimisation was conducted to identify the simplest combination of trial conditions related to a successful outcome. Coverage scores indicated the proportion of cases (i.e., trials) associated with the configuration where unique coverage scores indicated the proportion of cases that were only associated with the configuration. An overall coverage score of 1 indicates that all trials in the set were explained.

3) *Rigour tests:* To further simplify the minimised solution, logical remainders (configurations which are not represented in the existing trials) were then incorporated into the solution (analogous to imputation in statistical analysis).
  3a *Logical remainders:* First, the parsimonious solution was tested in which logical remainders were incorporated into the minimisation of the solution; this solution prioritised the simplicity of the expression over theoretical knowledge, with the software identifying the expected outcomes regardless of whether these were supported by the theory.
  3b *Intermediate solution:* Next, an intermediate solution was developed which incorporated logical remainders based on the reviewers’ assumptions of the directional expectations of the conditions in the set. The directional expectation was based on results from the Truth Tables and practical and theoretical knowledge. Additional checks were undertaken to determine the extent to which the treatment of logical remainders then represented contradictory simplifying assumptions following the practice of Dusa [22].

4) *Negated solution:* If the theory withstood any logical remainder and contradictory simplifying assumption tests, the inverse theory was then analysed by the negated outcome to check if the opposite theory was a better theory (i.e. had higher inclusion and consistency scores). If the PRI score is zero, in a negated solution, it indicates that the conditions identified are sufficient or enough to be associated with falls prevention.

5) *Final theory:* Finally, the identified theory was interpreted in the context of the case and theoretical knowledge to further determine its plausibility.

### Subgroup meta-analysis

The final QCA theory of configurations of critical conditions identified by QCA were tested in subgroup meta-analyses, to see if these drivers of outcomes explained the considerable heterogeneity observed in the meta-analysis of exercise trials in RACFs with falls measured at the end of the intervention period by Dyer and colleagues [10]. This provided estimates of the effectiveness of trials according to their alignment with the QCA theory. This method of using QCA to develop a theoretically driven subgroup analysis has been previously applied by Harris and colleagues [15] and Suen and colleagues, who applied the final QCA theory as a subgroup meta-analyses [25]. Subgroup meta-analyses were conducted using the generic inverse variance method using RevMan Web as described in the 2018 Cochrane Collaboration review [5]. Analyses were conducted for both rate of falls and risk of falling. Rate of falling was reported as a rate ratio and 95% confidence interval (CI). Risk of falling was reported as risk ratio and 95% CI.

### Certainty of the evidence

Two reviewers independently assessed the certainty of the evidence from the subgroup meta-analysis findings based on the GRADE approach, considering risk of bias, inconsistency, indirectness, imprecision, and other biases. The Cochrane risk of bias criteria previously reported [10], was used to consider risk of bias of the included studies for the GRADE approach [26].

## Results

**Fig. 2:**
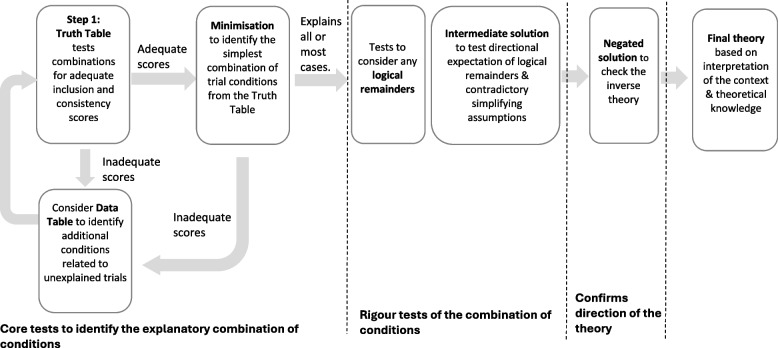
PRISMA flow diagram of systematic study selection [27]

Eighteen trials met the inclusion criteria for the QCA (Fig. 2). Examples of key studies excluded at full text review are given in Appendix 1a. Rate ratio outcomes data was used for fifteen trials and risk ratio was used for three trials to code outcomes (Table 1). The findings represent trials conducted in 11 countries enrolling 2,287 participants living in RACFs (Table 1). Participants were predominately ambulant females in their 70 s or 80 s with a degree of cognitive impairment who were mostly provided fully or initially supervised exercise programs (Table 1; Appendix 2 Table S1). Most trials (13 of 18) had scores that demonstrated ‘good’ quality based on the PEDro scale, whilst the remaining were ‘fair’ quality (Appendix 2, Table S1) [21].

**Table 1:** Study characteristics

Author, year	Study Design	Country	N randomised	Age, mean (SD)	Female (%)	Intervention	Control	Falls Outcome Used
Arrieta, 2019 [28]	RCT	Spain	112 I: 57 C: 55	84.9 (6.9) I: 85.1 (7.6) C: 84.7 (6.1)	70.5	Exercise professional supervised group training of individualised progressive multicomponent strength and balance exercises at moderate intensity. 1 h session twice a week for 6 months. Individually tailored walking recommendations based on baseline 6-min walking test performance and goal of completing 140 min per week for 6 months	Routine low intensity activities usual offered such as memory workshops, reading, singing, soft gymnastics etc	Rate of falls (Rate ratio, 95%CI): 0.45 (0.29, 0.69)
Brett, 2021 [29]	RCT	Australia	60 I A: 20 I B: 20 C: 20	85 (NR) I A: 86 (NR) I B: 84 (NR) C: 86 (NR)	66	Group exercises (a range of seated and standing exercises which targeted strength, balance, endurance, and flexibility) supervised by physiotherapist Intervention A: 45 min of group exercise per week once a week for 3 months Intervention B: 15 min 3 times a week for 3 months	Usual low intensity group activities such as seated bingo, concerts, movies, quizzes, and gentle range of movement exercises were encouraged. These activities were available for 30-60 min, once or twice a week	Rate of falls (Rate ratio, 95%CI): 0.23 (0.14,0.37)
Buckinx, 2014 [30]	RCT	Belgium	62 I: 31 C: 31	83.2 (7.9) I: 82.2 (9.0) C: 84.2 (6.8)	76	Physiotherapist or researcher supervised whole body vibration program (75 s or 1.25 min), three times a week with a minimum of 1 day between sessions for 6 months	Usual: no change to lifestyle of involvement in any new type of physical activity	Rate of falls (Rate ratio, 95%CI): 1.34 (0.73, 2.45)
Cadore, 2014 [31]	RCT	Finland	32 I: 16 C: 16	91.9 (4.1)	70	Supervised multicomponent exercises (i.e., warm up exercise, muscle power training, balance and gait retraining, and cool down exercise) for 40 min, twice weekly with 2 consecutive days between sessions for 3 months	Routine mobility exercises for 40 min at least 4 days a week, routinely encouraged in most Spanish nursing home	Rate of falls: no falls in intervention vs. 0.8 falls per patient per month (*p* < 0.001)
Choi, 2005 [32]	Quasi RCT	Korea	68 (recruited) I: 29 C: 30	77.9 (7.3)^a^ I: 77.0 (7.7) C: 78.7 (6.9)	75	35 min supervised Tai Chi group sessions, 3 times per week for 3 months	Usual routine activities	Number of fallers (Risk ratio, 95%CI): 0.60 (0.19, 1.87)
Dhargave, 2020 [33]	RCT	India	163 I: 82 C: 81	74.6 (8.5) I: 75.3 (8.7) C: 73.9 (8.3)	53.3	Program consisted of bilateral range of motive exercises and stretching as well as progressed bilateral strengthening and balance exercises. 30-min sessions once a day. Supervised by therapist for first week then visited once every 15 days for 3 months. Advised to also walk for 30 min in a day outside of the home	Falls prevention education program at the beginning of the study about identifying risk factors for falls, identifying, and avoiding environmental hazards, maintaining habit of at least 15 min of walking daily	Rate of falls (Rate ratio, 95%CI): 0.72 (0.44, 1.17)
Hewitt, 2018 [1]	Cluster RCT	Australia	221 I: 113 C: 108	86 (7.0)^a^ I: 86 (7) C: 87 (7)	65	Physiotherapist individually prescribed progressive resistance training plus balance exercises performed in a group setting for a total of 50 h over 25 weeks. Followed by a 6-month maintenance program twice weekly for 30 min by trained facility staff or volunteers	Continued with usual regular activity schedule without the intervention program	Rate of falls (Rate ratio, 95%CI): 0.45 (0.33, 0.61)
Irez, 2011 [34]	RCT	Turkey	60 I: 30 C: 30	75.4 (6.7)^a^ I: 72.8 (6.7) C: 78.0 (5.7)	75.4	60-min supervised Pilates sessions, 3 days per week for 3 months. For 1st 4 weeks mat exercises. After 4 weeks, additional Thera-Band elastic resistance exercises followed by Pilates ball exercises for beginners	Usual exercise. Told to refrain from new exercise programs or changing current level of exercise	Rate of falls (Rate ratio, 95%CI): 0.28 (0.15, 0.54)
Jahanpeyma, 2020 [35]	RCT	Turkey	72 I: 36 C: 36	75.2 (5.2)^a^ I: 74.6 (5.9) C: 75.8 (4.5)	73.6^a^	Researcher supervised small group (9 participants) Otago exercises for 45 min sessions 3 days a week for 1 month. Followed by performing exercise individually for 2 months where research visited weekly	Walking on level ground at normal pace for 30 min 3 days a week for 3 months. Demonstration once and given training and follow up booklet where researcher conducted weekly follow up and consultation in first month	Rate of falls (Rate ratio, 95%CI): 0.39 (0.23, 0.66)
Kovacs, 2013 [36]	RCT	Hungary	86 I: 43 C: 43	77.8 (11.3)^a^ I: 76.4 (9.6) C: 79.2 (12.7)	81	Physiotherapist supervised multimodal small group (2–4 participants) exercise program (based on Otago and included progressive resistance, strength, and balance) twice a week for 12 months. Supervised walking to practice gait elements once a week	No exercise program but participation in social activities	Rate of falls (Rate ratio, 95%CI): 0.77 (0.37, 1.62)
Mulrow, 1994 [37]	RCT	USA	194 I:97 C:97	Total: 80.6 ( 8.2)^a^ I: 79.7 (8.5) C: 81.4 (7.9)	71	Physical therapist tailored one-to-one exercises (gait, balance, coordination, strength/resistance, and flexibility) for 30 to 45 min, 3 times a week for 4 months	Friendly visit	Rate of falls (Rate ratio, 95%CI): 1.32 (0.95, 1.85)
Rosendahl, 2008 [38]	Cluster RCT	Sweden	191 I: 91 C: 100	84.7 (6.5) I: 85.3 (6.1) C: 84.2 (6.8)	73	Five 45-min sessions held every two weeks for 3 months. Functional exercise (e.g., weight-bearing challenging leg strength, postural stability, and gait ability) every fortnight, selected for each resident by physiotherapist. High intensity and increased load were encouraged	5 sessions of 45 min seated programme by occupational therapist including watching films, reading, and singing, every fortnight	Number of fallers (Risk ratio, 95%CI): 0.98 (0.69 to 1.39)
Sakamoto, 2006 [39]	RCT	Japan	553^b^	81.6 (9.0) I: 81.2 (NR) C: 82.3 (NR)	74	Standing unipedal balance exercise for both legs, for 1 min on each leg, 3 times a day for 6 months, led by physiotherapist	Usual care without exercise	Rate of falls (Rate ratio, 95%CI): 0.82 (0.65, 1.04)
Schoenfelder, 2000 [40]	RCT	USA	16 I: 9 C: 7	82.8 (NR)	75	20 min exercise, 3 times a week for 3 months, delivered by a research member. Supervised ankle strengthening exercises and up to 10 min of walking	Usual care	Rate of falls (Rate ratio, 95%CI): 2.86 (1.16, 7.04)
Shimada, 2004 [41]	RCT	Japan	32 I: 18 C: 14	Total: 82.4 (6.1)^a^ I: 81.8 (5.9) C: 83.1 (6.4)	78	Perturbed gait exercise on a treadmill (i.e., gait, balance, coordination, and endurance) supervised and individually tailored by physical therapists in additional to usual exercises. Total of 600 min of exercise over 6 months, 1 to 3 times per week	Usual exercise program including stretching, resistance training, group training and outdoor gait training	Rate of falls (33.3% in intervention vs. 54.6% in control, *p* = 0.426)
Toots, 2019 [42]	RCT	Sweden	186 I: 93 C: 93	85.1 (7.1) I: 84.4 (6.2) C: 85.9 (7.8)	76	Physiotherapist led group exercise consisting of 39 functional exercises at high intensity and in weight bearing positions similar to daily activities. 5 sessions per fortnight for 4 months	Attention control consisting of structured activities developed by occupational therapist (OT) or OT assistants such as seated group conversations, singing, listening to music, reading, or looking at pictures and objects	Rate of falls (Rate ratio, 95%CI): 1.30 (0.81, 2.08)
Varela, 2018 [43]	RCT	Spain	74 (39 completed) C:22 I: 17)	Total: 81.1 (8.2)^a^ I: 77.9 (8.8) C: 83.6 (7.1)	38^a^	Cycling at self-selected intensity for at least 15 min per day for 15 months	Recreational activities for 15 months	Rate of falls (Rate ratio, 95%CI): 0.67 (0.37, 1.21)
Yokoi, 2015 [44]	Cluster RCT	Japan	105 I: 51 C: 54	79.3 (6.7)^a^ I: 80.2 (7.9) C: 78.5 (5.2)	60	Six group-based supervised, seated short stick activities with rolled newspaper as the stick including a warm-up. 25 min per session, twice a week for 6 months	Usual activities including daily housekeeping, hobbies, work and 10-min group stretching exercises	Number of fallers (Risk ratio, 95%CI): 0.21 (0.03, 1.55)

*Abbreviations:* *C* Control, *I* Intervention, *NR* Not reported

^a^ Reviewer calculated

^b^ Number of participants for intervention and control group not reported

The included trials varied in terms of their characteristics, including the level of cognitive impairment of participants, degree of participant mobility, type and duration of exercise programs and study quality (Appendix 2, Table S1 and S2). This variation provides trial characteristic data suited to analysis with QCA [45, 46]. Specifically, five trials included only residents with no cognitive impairment while three trials included only residents with cognitive impairment; the remainder included a mix (Appendix 2, Table S1). The ability of participants to mobilise also varied with 11 trials mainly recruiting completely independent residents, 3 trials enrolling a majority of residents requiring walking aids to mobilise and 4 trials where the majority of residents required significant assistance to mobilise (Appendix 2, Table S1). Exercise interventions were provided for 3, 4, 6, 12 or 15 months in comparison to other non-exercise activities or usual care, amongst predominately groups of less than 50 participants in the control and intervention arms (Table 1). Two trials provided resident education on falls prevention in addition to exercise (Appendix 2, Table S1).

### Qualitative comparative analysis

**Fig. 3:**
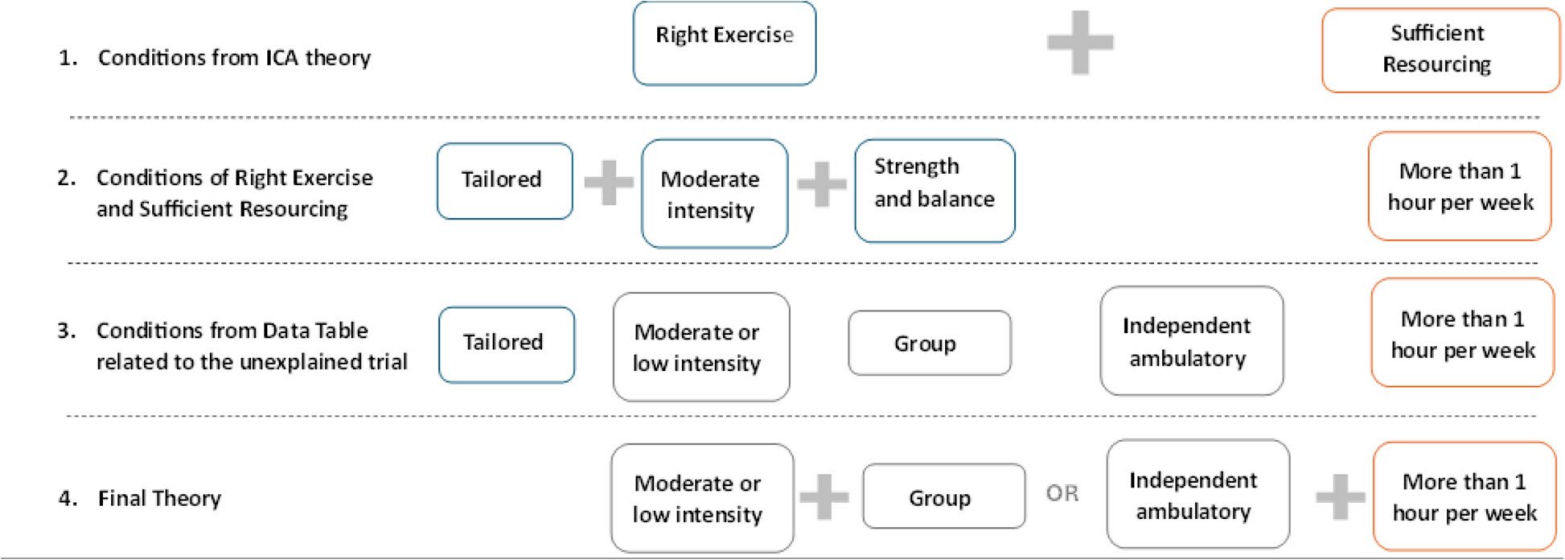
Summary of key conditions explored in QCA

Figure 3 provides a summary of the key conditions explored in each step of the QCA analysis.

#### Investigation of original theory from ICA

Providing the “right exercise” plus “sufficient resourcing” explained outcomes in 12 of 18 included trials (successful cases in configuration 1 and unsuccessful cases in configuration 4) (Table 2). This theory was not consistent with all trial outcomes as one trial which had both right exercise and sufficient resourcing was unsuccessful [42]; two trials with right exercise alone were successful [31, 41], one trial with sufficient resourcing alone was successful [43] and two trials with neither right exercise nor resourcing were successful [32, 44] (Table 2). As the presence or absence of right exercise alone was consistent with the outcomes in 14 of 18 of the included studies, the components of right exercise were further explored.

**Table 2:** Truth table of right exercise & sufficient resourcing

Configuration	Right exercise	Sufficient Resourcing	Outcome (Reduced Falls)	No. trials	Sufficiency		Cases	Supports theory
					Inclusion Score (incl)	Consistency Score (PRI)		
**1**	Yes^b^	Yes^b^	Yes	8	0.908	0.908	**Arrieta **[28]**, Brett **[29]**, Dhargave **[33]**, Hewitt **[1]**, Irez **[34]**, Jahanpeyma **[35]**, Kovacs **[36]**,** Toots [42]	Unclear
**2**	No	Yes	No	1	0.786	0.786	**Varela **[43]	No
**3**	Yes^b^	No^a^	No	2	0.667	0.667	**Cadore **[31]**, Shimada **[41]	No
**4**	No^a^	No^a^	No	7	0.349	0.349	**Choi **[32]**,** Buckinx [30], Sakamoto [39], Mulrow[37], Rosendahl [38], Schoenfelder [40], **Yokoi** [44]	Unclear

In QCA as ^a ^0, ^b ^1

Inclusion cut-off = 0.80 for fuzzy scoring

Right exercise was calculated by the addition of codes for Progressive standing strength and dynamic balance, Tailored exercise and Moderate intensity divided by 3

**Successful case**, unsuccessful case

#### Adaption 1 – Exploration of conditions of right exercise and sufficient resourcing

The components of the “right exercise” theory for reducing falls amongst residents in RACFs were: (i) progressive standing strength and dynamic balance exercise, which is (ii) tailored to the individual resident and (iii) conducted at moderate intensity [13]. The Truth Table examining these three conditions against trial outcomes was constructed (Appendix 3, Table S1). The presence of all three conditions explained falls reduction at the end of intervention period in 8 of 18 included trials (configuration 1) and the absence of all 3 components led to an unsuccessful outcome in 6 trials (configurations 3, 5 and 6) but did not explain the remaining 4 successful trials [32, 41, 43, 44] (in configurations 2, 3 and 4, Appendix 3, Table S1). Also, as progressive standing strength and dynamic balance exercise were present in both configurations that supported and did not support the theory, this condition did not appear to be a critical factor for trial outcomes.

Minimisation suggested that the combination of tailored exercise at moderate intensity was associated with falls reduction in 15 of 18 trials, with inclusion score of 1.000, proportional reduction inconsistency score 1.000 and coverage score of 0.750 (Appendix 3, Table S2).

Further examining this condition of the ICA theory, the dose of 30 h was derived from a trial testing 30 h over 25 weeks, i.e. a weekly dose of 1.2 h per week, which was tested in QCA as presence of more than 1 h per week [1]. The definition for ‘sufficient resourcing’ was then explored to identify condition/s that could be combined with moderate intensity and tailored.

A Truth Table of moderate intensity, tailored, and dose (more than 1 h per week) explained 17 out of 18 trials (Appendix 3, Table S3). Yokoi and colleagues trial was the only trial which did not provide tailored, moderate intensity exercise at this dose which was successful at reducing falls [44]. Conditions of this trial in the Data Table (Appendix 2, Table S1 & S2) were then considered for possible explanatory conditions explaining the trial’s outcomes.

#### Adaption 2: Conditions from the data table related to the unexplained trial with the theory from adaption 1

Enrolling only independent ambulatory participants, conducting exercise in a group, or conducting exercise at low intensity were conditions that appeared relevant for further investigation. Therefore, a Truth Table of only independent ambulatory participants, tailored and moderate/low intensity were considered together, providing an explanation for all 18 trials (Appendix 2, Table S4). Many configurations only explained one case with a solution that had too many conditions, creating 21 logical remainders, so further refinement through minimisation was required. In an intermediate solution where the logical remainders were analysed with a directional expectation that tailored, moderate/low intensity and dose was sufficient (based on Table S4 and considering Yokoi provided low intensity exercise). This intermediate solution offered two pathways for explaining success with the minimisation, suggesting that ‘tailored’ was not a differentiating driver in this set (Appendix 2, Table S5).

#### Final theory

A Truth Table of independent ambulatory participants, moderate or low intensity, group exercise and an exercise dose of more than 1 h per week demonstrated that these conditions were able to discriminate between successful and unsuccessful trials (Table 3). Minimisation demonstrated three scenarios that explained the success and failure of all 18 falls prevention exercise interventions in RACFs included in this sample, with the highest possible QCA inclusion and coverage scores of 1.0 (Table 4). The intermediate solution considering logical remainders with the directional expectation of the presence of moderate/low intensity and dose being the drivers, led to two solutions (Table 5). This did not have any contradictory simplifying assumptions. The negated solution also did not provide a better theory (Appendix 2, Table S6). Therefore, the solution posed as the final refined theory from the QCA was: Moderate or low intensity group exercise OR exercise for more than 1 h per week in independent ambulatory residents are the critical conditions of successful exercise programs for falls prevention in RACFs.

**Table 3:** Truth table of moderate or low intensity exercise, group, independent ambulatory residents and > 1 h per week

Configuration	Independent ambulatory	Moderate or low intensity	Group	More than 1 h per week	Outcome (Reduced Falls)	No. trials	Sufficiency		Cases
							Inclusion Score (incl)	Consistency Score(PRI)	
**1**	Yes	Yes	Yes	Yes	Yes	3	1.000	1.000	**Arrieta **[28]**, Jahanpeyma **[35]**, Kovacs **[36]
**2**	Yes	Yes	No	Yes	Yes	4	1.000	1.000	**Cadore **[31]**, Dhargave **[33]**, Shimada **[41], **Varela** [43]
**5**	Yes	Yes	Yes	Yes	Yes	1	1.000	1.000	**Hewitt **[1]**, Irez **[34]
**6**	Yes	No	Yes	Yes	Yes	1	1.000	1.000	**Choi **[32]
**7**	No	Yes	Yes	No	Yes	1	1.000	1.000	**Brett **[29]
**8**	Yes	Yes	Yes	No	Yes	1	1.000	1.000	**Yokoi **[44]
**11**	No	No	Yes	Yes	No	2	0.000	0.000	Rosendahl [38], Toots [42]
**3**	No	Yes	No	No	No	1	0.000	0.000	Buckinx [30], Sakamoto [39]
**4**	No	Yes	No	Yes	No	1	0.000	0.000	Mulrow[37]
**9**	Yes	Yes	No	No	No	1	0.000	0.000	Schoenfelder [40]

Inclusion set to 0.9

**Successful case**, unsuccessful case

**Table 4:** QCA minimisation of moderate or low intensity exercise, group, independent ambulatory residents and > 1 h per week

Configuration	Conditions			Outcome (Reduced Falls)	No. of successful studies	Sufficiency				Cases explained
						Inclusion Score (InclS)	Consistency Score (PRI)	Coverage (covS)	Unique Coverage (covU)	
**1**	Moderate/ Low Intensity		Group	Yes	7	1.000	1.000	0.583	0.333	**Brett **[29]**, Hewitt **[1]**, Irez **[34]**, Yokoi **[44]**, Arrieta **[28]**, Jahanpeyma **[35]**, Kovacs **[36]**,** Mulrow [37], Buckinx [30], Rosendahl [38], Sakamoto [39], Toots [42]
**2**	Independent ambulatory participants^a^	Moderate/ Low Intensity	More than 1 h per week	Yes	7	1.000	1.000	0.583	0.333	**Cadore **[31]**, Dhargave **[33]**, Shimada **[41], **Varela** [43]**, Arrieta **[28]**, Jahanpeyma **[35]**, Kovacs **[36]
**3**	Independent ambulatory participants^a^	Group	More than 1 h per week	Yes	4	1.000	1.000	0.333	0.083	**Choi **[32]**, Arrieta **[28]**, Jahanpeyma **[35]**, Kovacs **[36]**,** Schoenfelder [40],
**Overall**						**1.0000**	**1.000**	**1.000**		18

**Successful case**, unsuccessful case

^a^Independent ambulatory participants (independent with or without walking aid)

**Table 5:** QCA minimisation of Table 4 considering logical remainders and no contradictory assumptions

**Configuration**	**Conditions**		**Outcome (Reduced Falls)**	**No. of successful studies**	**Sufficiency**				**Cases explained**
					Inclusion Score (InclS)	Consistency Score (PRI)	Coverage (covS)	Unique Coverage (covU)	
**1**	Moderate/ Low Intensity	Group	Yes	7	1.000	1.000	0.583	0.333	**Brett **[29]**, Hewitt **[1]**, Irez **[34]**, Yokoi **[44]**, Arrieta **[28]**, Jahanpeyma **[35]**, Kovacs **[36] Buckinx[30], Rosendahl [38], Sakamoto [39], Toots [42], Mulrow [37]
**2**	Independent ambulatory participants^a^	More than 1 h per week	Yes	7	1.000	1.000	0.667	0.417	**Choi **[32]**, Cadore **[31]**, Dhargave **[33]**, Shimada **[41], **Varela **[43]**, Arrieta **[28]**, Jahanpeyma **[35]**, Kovacs **[36]**,** Schoenfelder [40]
**Overall**					**1.0000**	**1.000**	**1.000**	-	18

**Successful case**, unsuccessful case

^a^ Independent ambulatory participants (independent with or without walking aid)

### Top up search

The top up search identified three falls prevention exercise trials in RACFs that met the inclusion criteria (Appendix 1b). A three-arm trial provided moderate intensity group exercises (either calisthenics or multicomponent exercise) compared to usual care to residents over 12 months [47]. Both interventions reduced the rate of falls, supporting the final QCA theory [47]. Another trial providing moderate intensity group exercise was unsuccessful at preventing falls as it was not able to provide continuous exercise during the intervention period due to COVID-19 [48], which was identified by the trialists and in previous meta-analyses as critical for successful falls prevention in RACFs [10]. The third trial provided moderate intensity group exercise for more than 1 h per week but was unsuccessful at preventing falls after the 12-week intervention [49]. The lack of success was most likely due to the combined effects of a small sample size (*n* = 12 in each arm) that caused the trial to be unable to demonstrate its full effect and a small number of falls (4 falls in each arm), as mentioned by the trialist [49]. This trial therefore did not offer additional nuances to the final QCA the.

### Sub-group meta-analyses

**Fig. 4:**
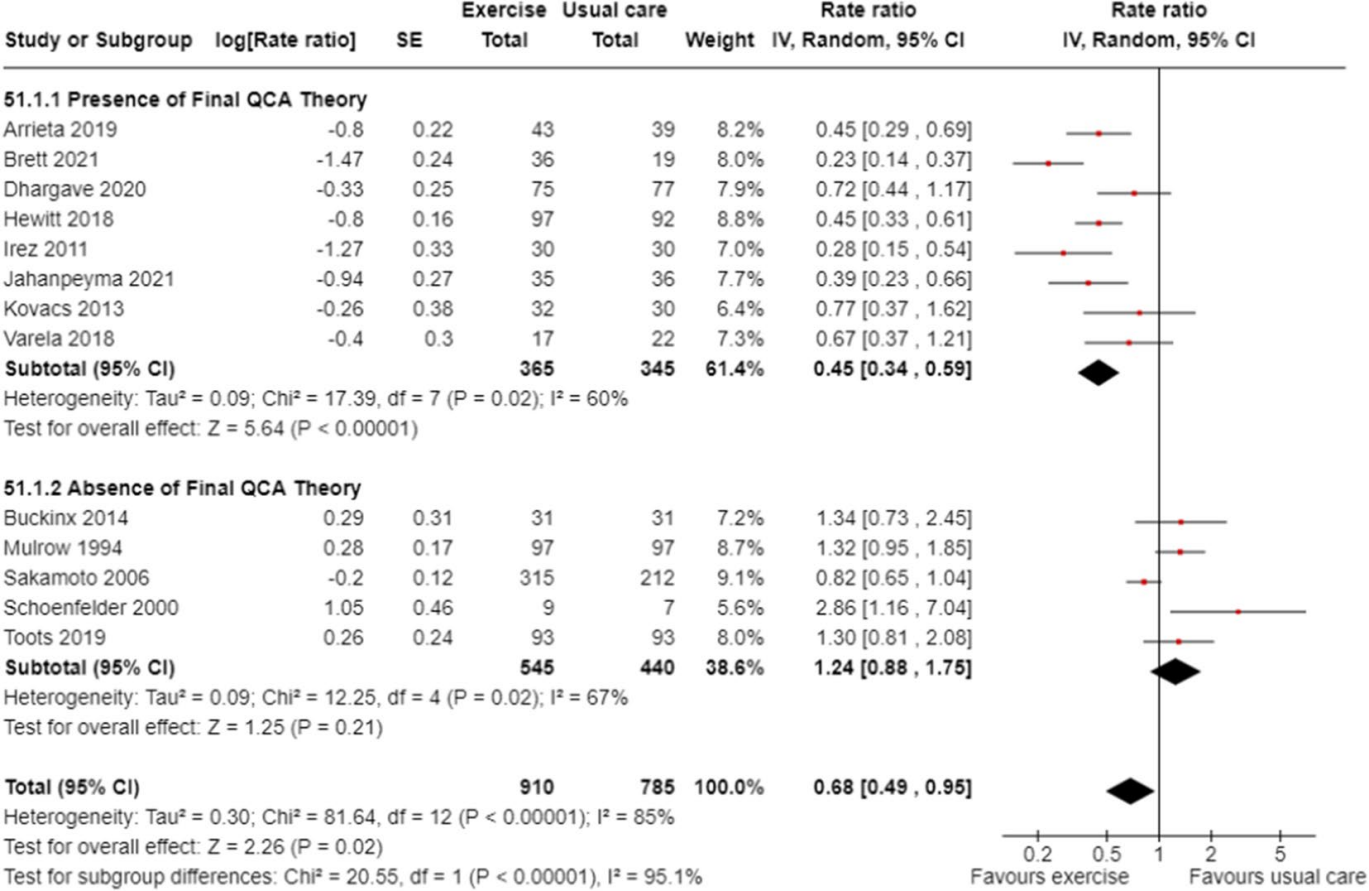
Meta-analysis of exercise in RACF grouped by the presence or absence of the final QCA theory ^a ^: rate of falls ^a^Moderate/low intensity group exercise for independent ambulatory residents for >1 hour per week

**Fig. 5:**
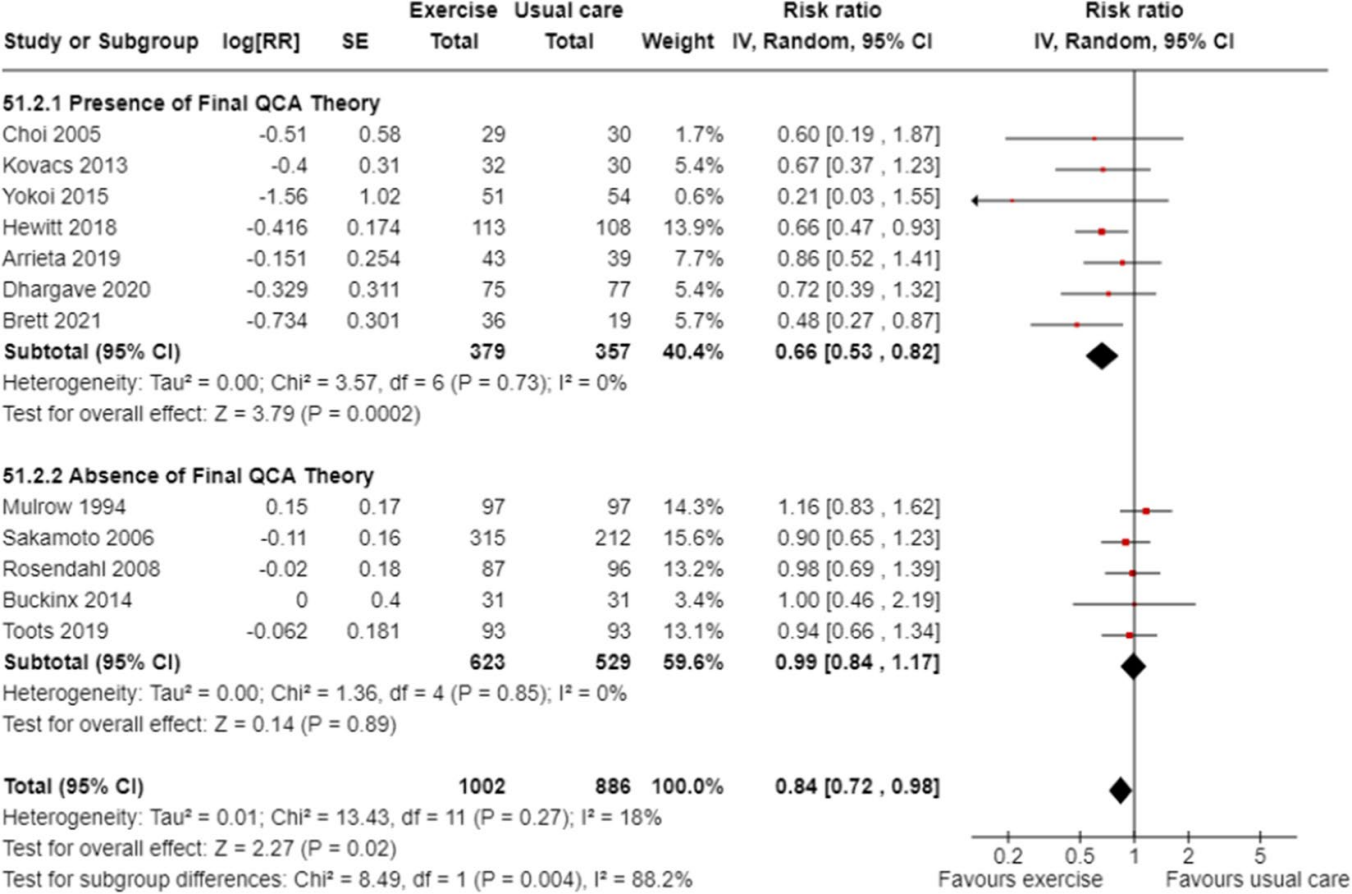
Meta-analysis of exercise in RACF grouped by the presence or absence of the final QCA theory ^a^ : risk of falling ^a^Moderate/low intensity group exercise for independent ambulatory residents for >1 hour per week

Sub-group meta-analyses confirmed greater fall prevention effects in trials that included the components of the final QCA theory (test for subgroup differences *p* < 0.01). Trials that had components aligning with the QCA final theory had a 55% reduction in the rate of falls with a reduction of the heterogeneity to moderate (Fig. 4, Rate Ratio 0.45, 95%CI 0.34 to 0.59, 8 trials, I^2^ = 60%) while the sub-group of trials without these conditions did not prevent falls (Fig.4, Rate Ratio 1.24, 95%CI 0.88 to 1.75, 5 trials, I^2^ = 67%). The final theory was also associated with a 34% reduction in the risk of falling (Fig. 5, Risk Ratio 0.66, 95%CI 0.53 to 0.82; 7 trials, I^2^ = 0%) while the sub-group of trials without these conditions did not prevent falls (Fig. 5, Risk Ratio 0.99, 95%CI 0.84 to 1.17, 5 trials, I^2^ = 0%). This provides low certainty evidence that group exercise conducted at a moderate intensity, or exercise for greater than one hour per week in ambulatory residents reduces falls in RACFs (Appendix 4).

### Discussion

This QCA of falls prevention trials in RACFs suggests that exercise interventions that provide moderate or low intensity group exercise, or exercise for more than 1 h per week in mostly independent ambulatory residents (who can independently mobilise with or without a walking aid), are more consistently associated with a reduction in falls. Moderate or low intensity exercise as a critical factor in exercise programs for residents suggests that high intensity exercise is not suitable for falls prevention in this setting [38, 42]. Most of the included successful trials tested a moderate intensity intervention (Table 1). Only three successful trials that mostly enrolled independent ambulatory residents conducted exercises at low intensity [32, 37, 43]. Thus, moderate intensity exercise is considered the most suitable approach for the broader range of exercise participants in RACFs and is consistent with the recommendation for “moderate challenge” in community falls prevention guidelines [4, 50–52].

Recently, a meta-analysis demonstrated that the effectiveness of exercise for falls prevention in this setting is not sustained beyond the intervention period [10]. The current study adds that a dose of more than 1 h per week of exercise for independent ambulatory residents is needed. The eight successful trials explained by this condition provided an average weekly exercise dose of 80 to 210 min [28, 31–33, 35, 36, 41, 43]. Only one trial [33] explained by this condition met the Australian physical activity guidelines for people aged 65 years and over of providing an average of 30 min of daily exercise per day during the intervention [53]. The remaining 10 trials provided a dose of 80 to 135 min per week, while the unsuccessful trial explained by this condition provided an average exercise dose of 51 min per week. This suggests that a dose of at least 80 min per week could be enough to offer benefit in this population and be feasible for conducting exercise falls prevention in RACFs where there is the additional need for environmental facilitators to guide action and self-efficacy amongst this frail group of older adults [54, 55].

Although moderate or low intensity exercise delivered in a group for all residents, or more than 1 h for ambulant residents, were the critical conditions that differentiated successful trials from unsuccessful trials, it is important to consider that this is driven by the context of the existing trials in this analysis. Within the included studies, the majority of exercise interventions were supervised for the whole trial duration [1, 28–32, 34–42, 44] or in the first week then bi-monthly [33]. Only one trial did not provide supervised exercise, and this was conducted for residents who were able to walk independently without aids [43]. This suggests that any exercise program in RACFs should be supervised, as a necessary part of safe exercise provision [56].

While tailored exercise was present in the penultimate theory (Appendix 3,Table S4), it was not a condition of the final QCA theory (Table 5). This was due to tailoring being absent in the one trial which only included residents who were able to safely walk independently without aids and thus participate in a self-paced cycling intervention. Therefore, tailored exercise is also considered likely to be important amongst independent ambulatory residents who use walking aids [43]. Trials including participants with reduced mobility required the support and supervision of exercise professionals to tailor their exercise program (Table 1). This aligns with the intention of physiotherapy services [57].

This study was not able to identify a specific type of exercise program as differentiating successful from unsuccessful trials. However, given the common use of progressive strength and balance exercises amongst fall prevention trials and this type of exercise also identified in the ICA theory, progressive strength and balance exercise appears to be a suitable based on available evidence. Additionally, progressive strength and balance exercises are recommended in the community setting [2]. Similarly, in other studies that did not measure falls, both tailored multicomponent exercise and resistance exercise have demonstrated improvement in gait speed and sit-to-stand performance in RACFs [58, 59]. Nevertheless, further evidence is required to confirm a specific type of exercise for successful falls prevention in this population.

### Strengths and limitations

While these findings indicate critical conditions of successful fall prevention exercise programs in RACF, governments and organisations need to consider the abilities of older adults when applying these findings. The evidence from available trials is more representative of residents who can independently mobilise with or without a walking aid, as this restrictive inclusion criteria was applied in most trials (Table 1). Admission to RACFs has been associated with a decline in ambulatory ability due to the loss of intrinsic capacity associated with ageing [60]. This decline is further evidenced by the trend of residents admitted to RACFs with increasing disability and complexity of health conditions over the last two decades [61]. In Australia, the average age of those admitted to RACFs over 2021 to 2022 were 83 years for men and 85 years for women compared to predominately 70 to 80 years of participants in the included trials (Table 1) [62]. Consequently, most residents currently living in RACFs are likely to be frailer and older than the residents in many of the included trials. Thus, residents are likely to require tailored exercise at an individualised dose [63].

As QCA utilises qualitative methods to code all sources of published information associated with the trial, it enables broader consideration of details of delivery and context of interventions than more quantitative methods. However, this approach is still limited by the sources of information available. Hence studies that did not report a condition were coded as not providing this condition, incorporating a degree of assumption. The number of trials analysed (*n* = 18) was within the range of 10 to 50 recommended as ideal for QCA in this context [46]. Whilst three unsuccessful trials and one successful trial with fair methodological quality based on the PEDro scale were included in the analysis, the QCA findings demonstrated that study quality was not clearly differentiating successful trials from unsuccessful trials at preventing falls in RACFs (Appendix 2, Table S1). Whilst the search for included studies in the ICA and QCA was only conducted until December 2022, the top up search until May 2024 identified an additional three trials that did not offer additional evidence or nuances that would alter the conclusions.

### Future implications

Future studies should report in detail the degree of mobility of participating residents, the degree of tailoring and support provided in the exercise program and rates of adherence to the exercise program. These details were not consistently reported in the included trials but would assist in understanding the transferability of the trial programs into practice and in conducting additional QCA which may help to further understand the residual heterogeneity between trials observed.

### Conclusion

Exercise interventions for falls prevention in RACFs should focus on providing moderate or low intensity group exercise. For falls prevention interventions that mostly include residents who can mobilise independently with or without a walking aid, exercise for at least 80 min per week should be provided. Considering the level of frailty and limited mobility of many residents currently in RACFs, supervision and exercise tailoring are likely to be also necessary and an individualised dose may be required. Future trials should focus on examining exercise interventions for less mobile residents and report on the mobility of participants, degree of tailoring and support provided as well as adherence rates. When further data allows, future analyses should consider if there is a specific type of exercise that more successfully prevents falls in this population.

### Supplementary Information

Supplementary Material 1.

## Data Availability

Supplementary files contain all available data.
