# Supplementary material for: Qualitative Comparative Analysis of exercise interventions for fall prevention in residential aged care facilities
Source: BMC Geriatr. 2024 Sep 3;24:728. doi: 10.1186/s12877-024-05246-0 (PMC11370059; doi:10.1186/s12877-024-05246-0)
Supplement: Supplementary file 1 — Supplementary Material 1. [file 12877_2024_5246_MOESM1_ESM.docx]

**Appendices**

**Appendix 1: Top up search strategies**

| **Source** | **Search strategy** |
| --- | --- |
| 1. CENTRAL | #1 MeSH descriptor: [Accidental Falls] this term only  #2 MeSH descriptor: [Hip Fractures] explode all trees and with qualifier(s): [prevention & control - PC]  #3 (falls or faller$)  #4 #1 or #2 or #3  #5 MeSH descriptor: [Aged] explode all trees  #6 MeSH descriptor: [Middle Aged] this term only  #7 (older or senior$ or elderly)  #8 #5 or #6 or #7  #9 #4 and #8  #10 MeSH descriptor: [Residential Facilities] explode all trees  #11 MeSH descriptor: [Long-Term Care] this term only  #12 MeSH descriptor: [Institutionalization] this term only  #13 ((long stay or long term) NEAR/3 (care or ward*))  #14 (hostel* or nursing home* or care home* or care facilit* or rest home*)  #15 residen*  #16 institution*  #17 [65-#16]  #18 #9 and #17  Date added to CENTRAL trials database 07/12/2022-10/05/2024 140 |
| 2. MEDLINE | **1**  Accidental Falls/ or exp Hip Fractures/pc [Prevention & Control]  **2**  (falls or faller$).tw.  **3**  or/1-2  **4**  exp Aged/ or Middle Aged/  **5**  (older or senior$ or elderly).tw.  **6**  or/4-5  **7**  and/3,6  **8**  exp Residential Facilities/  **9**  Long-Term Care/  **10**  Institutionalization/  **11**  ((long stay or long term) adj3 (care or ward$1)).tw.  **12**  (hostel$1 or nursing home$ or care home$1 or care facilit* or rest home$).tw.  **13**  residen$.tw.  **14**  institution$.tw.  **15**  or/8-14  **16**  and/7,15  **17**  Randomized controlled trial.pt.  **18**  Controlled clinical trial.pt.  **19**  randomized.ab.  **20**  placebo.ab.  **21**  Drug therapy.fs.  **22**  randomly.ab.  **23**  trial.ab.  **24**  groups.ab.  **25**  or/17-24  **26**  exp Animals/ not Humans/  **27**  25 not 26  **28**  16 and 27  **29**  limit 28 to ed=20221207-20240510 |
| 3. EMBASE | **1**  Falling/ or exp Hip fracture/pc  **2**  (falls or faller$).tw.  **3**  1 or 2  **4**  Aged/ or Middle Aged/  **5**  (older or senior$ or elderly).tw.  **6**  4 or 5  **7**  3 and 6  **8**  exp residential home/  **9**  long term care/  **10**  institutionalization/  **11**  ((long stay or long term) adj3 (care or ward$1)).tw.  **12**  (hostel$1 or nursing home$ or care home$1 or care facilit* or rest home$).tw.  **13**  residen$.tw.  **14**  institution$.tw.  **15**  or/8-14  **16**  7 and 15  **17**  exp Randomized Controlled Trial/ or exp Single Blind Procedure/ or exp Double Blind Procedure/ or Crossover Procedure/  **18**  (random* or RCT or placebo or allocat* or crossover* or 'cross over' or trial or (doubl* adj1 blind*) or (singl* adj1 blind*)).ti,ab.  **19**  17 or 18  **20**  (exp Animal/ or animal.hw. or Nonhuman/) not (exp Human/ or Human cell/ or (human or humans).ti.)  **21**  19 not 20  **22**  16 and 21  **23**  limit 22 to dd=20221207-20240510  **24**  limit 22 to rd=20221207-20240510  **25**  23 or 24 |
| 4. CINAHL | S23 S16 AND S22 Limiters - Publication Date: 20221201-20240531  S22 S17 OR S18 OR S19 OR S20 OR S21  S21 TI random* OR AB random*  S20 TI ( (single blind* or double blind*) ) OR AB ( (single blind* or double blind*) )  S19 TI clinical trial* OR AB clinical trial*  S18 (MH "Clinical Trials+")  S17 PT Clinical Trial  S16 S7 AND S15  S15 S8 OR S9 OR S10 OR S11 OR S12 OR S13 OR S14  S14 TI institution* OR AB institution*  S13 TI residen* OR AB residen*  S12 TX (hostel* or nursing home* or care home* or care facilit* or rest home*)  S11 TX (long stay or long term) N3 (care or ward*)  S10 MH institutionalization  S9 (MH "Long Term Care")  S8 (MH "Residential Facilities+")  S7 S3 AND S6  S6 S4 OR S5  S5 TI ( (senior or seniors or elderly or older) ) OR AB ( (senior or seniors or elderly or older) )  S4 (MH "Aged+")  S3 S1 OR S2  S2 TI ( (falls or faller or fallers) ) OR AB ( (falls or faller or fallers) )  S1 (MH "Accidental Falls") |

**Appendix 1a: Key studies excluded at full text review.**

| **Source of Key studies excluded** | **Exclusion reason** | **Study citation** | **Reason for exclusion** |
| --- | --- | --- | --- |
| From included exercise vs. usual care studies in Cameron 2018 | Wrong outcome | Buettner, 2002 [66] | No suitable falls data as effect estimates and confidence interval or numerical falls data with p-values not provided |
|  | Wrong outcome | Da Silva Borges, 2014 [67] | No effect estimates and confidence interval for falls data. While p-value is provided, data to indicate number of falls was not reported. |
|  | Wrong outcome | Toulotte, 2003 [68] | No effect estimates and confidence interval for falls data |
|  | Wrong outcome | Sihvonon 2004 [69, 70] | Falls data only at a follow up time point. No falls data at end of intervention. |
|  | Wrong outcome | Kerse 2008 [71] | Falls data only at a follow up time point. No falls data at end of intervention. |
|  | Wrong outcome | Faber 2006 [72] | Falls data only at a follow up time point. No falls data at end of intervention. |
| From search update | Ongoing study | Grede, 2021[73] | Walking programme for older adults living in the community or nursing homes. Protocol only. |
|  | Wrong outcome | Buhring 2019 [74] | Pilot study of semi-recumbent vibration exercise where falls were measured as adverse events. |
|  | Wrong outcome | Birimoglu Okuyan, 2021[75] | No effect estimates and confidence intervals for falls data |
|  | Wrong outcome | Taani, 2022 [76] | Pilot study of semi-recumbent vibration exercise where no falls data was measured. |
|  | Wrong study design | Zou, 2022[77] | Group based Otago exercise program in nursing homes, but participants self-selected allocation and therefore study design was not randomised. |

**Appendix 1b: Study characteristics of trials identified in top up search**

| **Author, year** | **Study Design** | **Country** | **N randomised** | **Age, mean (SD)** | **Female (%)** | **Intervention** | **Control** | **Falls Outcome Used** |
| --- | --- | --- | --- | --- | --- | --- | --- | --- |
| Taylor, 2024 [48] | RCT | New Zealand | 520  I: 262  C: 258 | 84 (8)  I: 83.99 (7.7)  C: 84.22(7.5 | NR | Progressive balance and strength exercise program group sessions for 1- hour, twice a week by a physical therapist for 6 months and progressed based on participants abilities. | Group seated activities with no resistance or progressions such as seated swimming, seated marching, heel, and toe tapping, seated activities such as ballon catch, and throw led by long term staff. | Rate of falls (rate ratio, 95%CI):  0.98 (0.76, 1.27) |
| Bays-Moneo, 2023 | RCT | Spain | 69 IA: 23 IB: 23 C: 23 | 87.7 (6.9)  IA: 98.6 (6.6)  IB: 90.3 (6.8)  C: 89.2 (7.3) | 72.5% | IA: Multicomponent moderate intensity group exercise program, with twice-weekly progressive resistance training combined with 3 days a week of balance and gain retraining for 12 months.  IB: Calisthenics exercise including warm up, resistance training and flexibility exercise, 5 sessions per week for 40minutes each in addition to moderate intensity gait retraining for 12 months. | Usual care provided by nursing home which included rehabilitation if necessary and recommendation of daily walking around the centre. | Rate of falls (rate ratio, 95%CI):  IA: 0 ^a^, 95%CI not calculable (0 falls in intervention arm) IB: 0.50 (0.05, 5.51) ^a^ |
| Sadaqa, 2024 | RCT | Hungary | 24  I: 12  C: 12 | NR  I:78.3(7.0) C:78.5 (7.4) | 70.8% ^a^ | Multicomponent moderate intensity group exercise consisting of strength, balance, and aerobic exercises, twice a week on non-consecutive days for 45 to 60 mins per session provided by physiotherapists working in the facility. Exercise was progressed based in individual abilities. | Usual care of routine low intensity activities usually offered by the facility. | Rate of falls (rate ratio,95%CI):  1.0 (0.25 to 4.0) ^a^ |

^a^ reviewer calculated.

Abbreviations: C: control; I: intervention; NR: not reported, RCT: randomised controlled trial

**Appendix 2: Data Tables & Analyses**

**Table S1: Original Data Table of Included Trials**

|  | **Study, year** | **Participant Characteristics** | | | | | **Design Characteristics** | | **Intervention Characteristics** | | | | **Implementation Characteristics** | | | | **Falls Outcomes** |
| --- | --- | --- | --- | --- | --- | --- | --- | --- | --- | --- | --- | --- | --- | --- | --- | --- | --- |
|  |  |  |  |  |  |  |  |  | **Components of Right Exercise** | | | **ICA part 1** | **Components of Supporting exercise engagement** | | | **ICA part 2** |  |
|  |  | All have CI^a^ | All do not have CI^a^ | Degree of CI ^a,b^ | Degree of Mobility ^c^ | Independent ambulatory only ^d^ | Study Quality ^e^ | Small Study Size ^f^ | PSSDB^g^ | Tailored ^a^ | Moderate Intensity ^a^ | Right Exercise ^h^ | Sufficient resourcing ^a^ | Group exercise ^a^ | Falls education ^a^ | Supporting exercise engagement ^i^ | Successful |
| 1 | Arrieta, 2019 [30] | 0 | 0 | 1 | 1 | 1 | 0.6 | 0 | 1 | 1 | 1 | 1 | 1 | 1 | 0 | 0.7 | 1 |
| 2 | Brett, 2021 [31] | 1 | 0 | 0 | 0.3 | 0 | 0.6 | 1 | 1 | 1 | 1 | 1 | 1 | 1 | 0 | 0.7 | 1 |
| 3 | Buckinx 2014 [32] | 0 | 0 | 1 | 0.7 | 0 | 0.6 | 0 | 0.3 | 0 | 0 | 0.1 | 0 | 0 | 0 | 0 | 0 |
| 4 | Cadore 2014 [33] | 0 | 1 | 0 | 1 | 1 | 0.4 | 1 | 1 | 1 | 1 | 1 | 0 | 0 | 0 | 0 | 1 |
| 5 | Choi, 2005 [34] | 0 | 0 | 1 | 1 | 1 | 0.6 | 1 | 0.7 | 0 | 0 | 0 | 0 | 1 | 0 | 0.3 | 1 |
| 6 | Dhargave 2020 [35] | 0 | 0 | 1 | 1 | 1 | 0.6 | 0 | 1 | 1 | 1 | 1 | 1 | 0 | 1 | 0.7 | 1 |
| 7 | Hewitt 2018 [1] | 0 | 0 | 1 | 0.3 | 0 | 0.8 | 0 | 1 | 1 | 1 | 1 | 1 | 1 | 1 | 1 | 1 |
| 8 | Irez, 2011[36] | 0 | 0 | 1 | 1 | 1 | 0.6 | 0 | 0.6 | 1 | 1 | 0.9 | 1 | 1 | 0 | 0.7 | 1 |
| 9 | Jahanpeyma 2020 [37] | 0 | 1 | 0 | 1 | 1 | 0.6 | 0 | 1 | 1 | 1 | 1 | 1 | 1 | 1 | 1 | 1 |
| 10 | Kovacs 2013 [38] | 1 | 0 | 0 | 1 | 1 | 0.8 | 0 | 1 | 1 | 1 | 1 | 1 | 1 | 0 | 0.7 | 1 |
| 11 | Mulrow 1994 [39] | 0 | 0 | 1 | 0.3 | 0 | 0.6 | 0 | 0.3 | 1 | 0 | 0.4 | 0 | 0 | 0 | 0 | 0 |
| 12 | Rosendahl 2008 [40] | 0 | 0 | 1 | 0.7 | 1 | 0.7 | 0 | 1 | 1 | 0 | 0.3 | 0 | 1 | 0 | 0.3 | 0 |
| 13 | Sakamoto 2006 [41] | 0 | 0 | 1 | 0.7 | 1 | 0.4 | 0 | 0 | 0 | 0 | 0 | 0 | 0 | 0 | 0 | 0 |
| 14 | Schoenfelder 2000 [42] | 0 | 0 | 1 | 1 | 1 | 0.4 | 1 | 0.3 | 0 | 0 | 0.1 | 0 | 0 | 0 | 0 | 0 |
| 15 | Shimada 2004 [43] | 0 | 1 | 0 | 1 | 1 | 0.4 | 1 | 0.3 | 1 | 1 | 0.8 | 0 | 0 | 0 | 0 | 1 |
| 16 | Toots 2019 [44] | 1 | 0 | 0 | 0.3 | 0 | 0.8 | 0 | 1 | 1 | 0 | 0.7 | 1 | 1 | 0 | 0.7 | 0 |
| 17 | Varela 2018 [45] | 0 | 1 | 0 | 1 | 1 | 0.6 | 1 | 0 | 0 | 0 | 0 | 1 | 0 | 0 | 0.3 | 1 |
| 18 | Yokoi 2015 [46] | 0 | 1 | 0 | 1 | 1 | 0.7 | 1 | 0 | 0 | 0 | 0 | 0 | 1 | 0 | 0 | 1 |

Abbreviations: CI, cognitive impairment; ICA, intervention component analysis theory; PSSDB, Progressive standing strength and dynamic balance.

^a^ 1= yes, 0 = no

^b^ As all residents included in inclusion criteria

^c^ 1 = majority (over 50%) completely independent, no walking aids, 0.7 = majority (over 50%) ambulant with walking aids, 0.6= majority (over 50%) needing 1 assist to mobile, 0.3= majority needing significant assistance to mobilise

^d^ 1 = inclusion criteria only independent ambulatory residents

^e^ Study quality based on Physiotherapy Evidence Database (Pedro) Risk of Bias Tool score out of 10; divided by 10

Less than 30 participants in each trial arm.

^g^ 1= yes, 0 = no, 0.6 = mostly fulfills the component, 0.3 =partially fulfils the exercise component or involvement in the exercise or progression varies

^h^ Calculated by the average of scores for Progressive standing strength and dynamic balance, Tailored and Moderate intensity.

^i^ Calculated by the average of scores for ‘Sufficient resourcing’, ‘Group exercise’ and ‘falls education’.

**Table S2: Additional conditions added to Data Table to refine the theory in QCA**

| **Study, year** | | **Intervention Characteristics** | | | | | |  | |  |  |
| --- | --- | --- | --- | --- | --- | --- | --- | --- | --- | --- | --- |
|  |  |  |  |  |  |  |  |  | |  |  |
|  |  | Components of progressive standing strength and balance | | | Additional conditions related to “Right exercise” | | | Additional conditions related to “Sufficient Resourcing” | | | |
|  |  | Progressive Strength Training ^a^ | Progressive dynamic balance training ^a^ | Progressive Standing Exercise  ^a^ | Low intensity ^b^ | High intensity ^b^ | Moderate or low intensity ^b^ | Supervised exercise ^c^ | Dose of 30 hours ^b^ | Dose > 1 hour per week ^b^ | Funded ^b^ |
| 1 | Arrieta, 2019 [30] | 1 | 1 | 1 | 0 | 0 | 1 | 1 | 1 | 1 | 1 |
| 2 | Brett, 2021 [31] | 1 | 1 | 1 | 0 | 0 | 1 | 1 | 0 | 0 | 1 |
| 3 | Buckinx 2014 [32] | 0 | 0 | 1 | 1 | 0 | 1 | 1 | 0 | 0 | 0 |
| 4 | Cadore 2014 [33] | 1 | 1 | 1 | 0 | 0 | 1 | 1 | 0 | 1 | 1 |
| 5 | Choi, 2005 [34] | 0 | 1 | 1 | 1 | 0 | 1 | 1 | 0 | 1 | 0 |
| 6 | Dhargave 2020 [35] | 1 | 1 | 1 | 0 | 0 | 1 | 0.4 | 1 | 1 | 1 |
| 7 | Hewitt 2018 [1] | 1 | 1 | 1 | 0 | 0 | 1 | 1 | 1 | 1 | 1 |
| 8 | Irez, 2011[36] | 1 | 0.3 | 0.3 | 0 | 0 | 1 | 1 | 1 | 1 | 1 |
| 9 | Jahanpeyma 2020 [37] | 1 | 1 | 1 | 0 | 0 | 1 | 0.6 | 0 | 1 | 1 |
| 10 | Kovacs 2013 [38] | 1 | 1 | 1 | 0 | 0 | 1 | 1 | 1 | 1 | 1 |
| 11 | Mulrow 1994 [39] | 0.3 | 0.3 | 0.3 | 1 | 0 | 1 | 1 | 0 | 1 | 0 |
| 12 | Rosendahl 2008 [40] | 1 | 1 | 1 | 0 | 1 | 0 | 1 | 0 | 1 | 0 |
| 13 | Sakamoto 2006 [41] | 0 | 0 | 1 | 1 | 0 | 1 | 1 | 0 | 0 | 0 |
| 14 | Schoenfelder 2000 [42] | 0 | 0 | 1 | 1 | 0 | 1 | 1 | 0 | 0 | 0 |
| 15 | Shimada 2004 [43] | 0 | 0 | 1 | 0 | 0 | 1 | 1 | 0 | 1 | 0 |
| 16 | Toots 2019 [44] | 1 | 1 | 1 | 0 | 1 | 0 | 1 | 1 | 1 | 1 |
| 17 | Varela 2018 [45] | 0 | 0 | 0 | 1 | 0 | 1 | 0 | 0 | 1 | 1 |
| 18 | Yokoi 2015 [46] | 0 | 0 | 0 | 1 | 0 | 1 | 1 | 0 | 0 | 0 |

^a^ 1= yes, 0 = no, 0.6 = mostly fulfills the component, 0.3 =partially fulfils the exercise component or involvement in the exercise or progression varies

^b^ 1= yes, 0 = no

^c^1= yes, 0.6 = mostly supervised, 0.4 = partially supervised, 0 = no.

**Appendix 3: QCA**

**Table S1: Truth Table of Conditions of Right Exercise**

| **Configuration** | **Progressive standing strength & dynamic balance** | **Tailored** | **Moderate Intensity** | **Outcome (Reduced Falls)** | **No. studies** | **Sufficiency** | | **Cases** | **Supports theory** |
| --- | --- | --- | --- | --- | --- | --- | --- | --- | --- |
|  |  |  |  |  |  | Inclusion Score  (incl) | Consistency Score  (PRI) |  |  |
| **1** | Yes^b^ | Yes^b^ | Yes^b^ | Yes | 8 | 1.000 | 1.000 | **Arrieta [30], Brett [31], Cadore [33], Dhargave [35], Hewitt [1], Jahanpeyma [37], Kovacs [38], Irez [36]** | Yes |
| **2** | No^a^ | Yes^b^ | Yes^b^ | Yes | 1 | 1.000 | 1.000 | **Shimada [43]** | No |
| 3 | No^a^ | No^a^ | No^a^ | No | 5 | 0.523 | 0.523 | Buckinx [32], Sakamoto [41], Schoenfelder [42], **Varela [45], Yokoi [46]** | Unclear |
| **4** | Yes^b^ | No^a^ | No^a^ | No | 1 | 0.438 | 0.438 | **Choi [34]** | No |
| 5 | No^a^ | Yes^b^ | No^a^ | No | 1 | 0.000 | 0.000 | Mulrow [39] | Yes |
| 6 | Yes^b^ | Yes^b^ | No^a^ | No | 2 | 0.000 | 0.000 | Rosendahl [40], Toots [44] | Yes |

Coded in QCA as ^a^ 0, ^b^ 1,

Inclusion cut-off = 0.9

**Successful case,** unsuccessful case.

**Table S2: Minimisation of components of Right Exercise**

| **Configuration** | **Conditions** | | **Outcome (Reduced Falls)** | **No. of studies** | **Sufficiency** | | | **Cases explained** | **Cases unexplained** |
| --- | --- | --- | --- | --- | --- | --- | --- | --- | --- |
|  |  |  |  |  | Inclusion Score (InclS) | Consistency Score (PRI) | Coverage Score (covS) |  |  |
| **1** | Tailored exercise | Moderate intensity | Yes | 9 | 1.000 | 1.000 | 0.750 | **Arrieta [30], Brett [31], Cadore [33], Dhargave [35], Hewitt [1], Irez [36], Jahanpeyma [37], Kovacs [38], Shimada [43]** Buckinx [32], Mulrow [39], Rosendahl [40], Sakamoto[41], Schoenfelder[42], Toots [44] | **Choi [34], Varela [45], Yokoi [46]** |
| **Overall** |  |  |  |  | **1.0000** | **1.000** | **0.750** | **15** | **3** |

Inclusion cut-off = 0.9.
**Successful case,** unsuccessful case.

**TableS3: Adaption 1 Tailored, Moderate Intensity and >1 hour per week exercise**

| **Configuration** | **Tailored** | **Moderate intensity** | **Dose > 1 hour per week** | **Outcome (Reduced Falls)** | **No. studies** | **Sufficiency** | | **Cases** | **Supports theory** |
| --- | --- | --- | --- | --- | --- | --- | --- | --- | --- |
|  |  |  |  |  |  | Inclusion Score  (incl) | Consistency Score (PRI) |  |  |
| **1** | No | No | Yes | Yes | 2 | 1.000 | 1.000 | **Choi [34], Varela [45]** | Yes |
| **2** | Yes | Yes | No | Yes | 1 | 1.000 | 1.000 | **Brett [31]** | Yes |
| **3** | Yes | Yes | Yes | Yes | 8 | 1.000 | 1.000 | **Arrieta[30], Cadore[33], Dhargave [35], Hewitt[1], Irez [36], Jahanpeyma [37], Kovacs [38], Shimada [43],** | Yes |
| **4** | No | No | No | No | 4 | 0.250 | 0.250 | Buckinx [32], Sakamoto[41], Schoenfelder[42], **Yokoi [46]** | Unclear |
| **5** | Yes | No | Yes | No | 3 | 0.250 | 0.250 | Rosendahl [40], Toots [44], Mulrow [39] | Yes |

**Successful case,** unsuccessful case.

**Table S4: Truth Table of Adaption 2**

| **Configuration** | **Independent ambulatory participants** ^a^ | **Tailored** | **Moderate or low intensity** | **Group** | **Dose > 1 hour per week** | **Outcome (Reduced Falls)** | **No. studies** | **Sufficiency** | | **Cases** | **Supports theory** |
| --- | --- | --- | --- | --- | --- | --- | --- | --- | --- | --- | --- |
|  |  |  |  |  |  |  |  | Inclusion Score  (incl) | Consistency Score (PRI) |  |  |
| **1** | No | Yes | Yes | Yes | No | Yes | 1 | 1.000 | 1.000 | **Brett [31]** | Yes |
| **2** | No | Yes | Yes | Yes | Yes | Yes | 2 | 1.000 | 1.000 | **Hewitt[1], Irez [36]** | Yes |
| **3** | Yes | No | No | Yes | Yes | Yes | 1 | 1.000 | 1.000 | **Choi [34]** | Yes |
| **4** | Yes | No | Yes | No | Yes | Yes | 1 | 1.000 | 1.000 | **Varela [45]** | Yes |
| **5** | Yes | No | Yes | Yes | No | Yes | 1 | 1.000 | 1.000 | **Yokoi [46]** | Yes |
| 6 | Yes | Yes | Yes | No | Yes | Yes | 3 | 1.000 | 1.000 | **Cadore[33], Dhargave [35], Shimada [43]** | Yes |
| 7 | Yes | Yes | Yes | Yes | Yes | Yes | 3 | 1.000 | 1.000 | **Arrieta[30], , Jahanpeyma [37], Kovacs [38]** | Yes |
| 8 | No | No | Yes | No | No | No | 2 | 0.000 | 0.000 | Buckinx [32], Sakamoto[41] | Yes |
| 9 | No | Yes | No | Yes | Yes | No | 2 | 0.000 | 0.000 | Rosendahl [40], Toots [44] | Yes |
| 10 | No | Yes | Yes | No | Yes | No | 1 | 0.000 | 0.000 | Mulrow [39] | Yes |
| 11 | Yes | No | Yes | No | No | No | 1 | 0.000 | 0.000 | Schoenfelder[42] | Yes |

^a^ Independent ambulatory participants (independent with or without walking aid)

**Successful case,** unsuccessful case.

**Table S5: Intermediate solution* for Adaption 2**

| **Configuration (Possible Pathway)** | **Conditions** | | | **Outcome (Reduced Falls)** | **No. studies** | **Sufficiency** | | | | **Cases explained** |
| --- | --- | --- | --- | --- | --- | --- | --- | --- | --- | --- |
|  |  |  |  |  |  | Inclusion Score (InclS) | Consistency Score (PRI) | Coverage (covS) | Unique Coverage (covU) |  |
| **1** | Independent ambulatory participants ^a^ | Dose >1 hour per week | | Yes | 9 | 1.000 | 1.000 | 0.667 | 0.417 | **Choi [34], Varela [45], Cadore [33], Dhargave[35], Shimada [43]**, **Arrieta [30], Irez [36] , Kovacs [38], Jahanpeyma[37],** Schoenfelder [42] |
| **2** | Moderate/Low Intensity | Group | | Yes | 7 | 1.000 | 1.000 | 0.583 | 0.333 | **Brett [31], Hewitt [1], Irez [36], Yokoi [46],**  **Arrieta [30], Jahanpeyma [37], Kovacs [38],** Buckinx [32], Mulrow[39], Rosendahl [40], Toots [44], Sakamoto [41], Buckinx [32], |
| **Overall** |  |  |  |  |  | **1.0000** | **1.000** | **1.000** |  | **18** |

^a^ Independent ambulatory participants (independent with or without walking aid)

**Successful case,** unsuccessful case.
*Includes 21 logical remainders with the directional expectation of 0,1,1,0,1 for presence of tailored, moderate/low intensity and dose as driving trial outcomes

**Table S6: Negated Solution***

| **Configuration** | **Conditions** | | | | **Outcome (Reduced Falls)** | **No. unsuccessful studies** | **Sufficiency** | | | | **Ineffective cases explained** |
| --- | --- | --- | --- | --- | --- | --- | --- | --- | --- | --- | --- |
|  |  |  |  |  |  |  | Inclusion Score (InclS) | Consistency Score (PRI) | Coverage (covS) | Unique Coverage (covU) |  |
| **1** | Not independent ambulatory participants ^a^ | Moderate/Low Intensity | Group | | No | 2 | 1.000 | 1.000 | 0.500 | 0.167 | Sakamoto [41], Buckinx [32], Mulrow [39] |
| **2** | Moderate/Low Intensity |  | Individual | Dose of less than 1 hour per week | No | 1 | 1.000 | 1.000 | 0.500 | 0.167 | Schoenfelder [42] |
| **3** | Not independent ambulatory participants ^a^ | High Intensity | Group | Dose of more than 1 hour per week | No | 2 | 1.000 | 1.000 | 0.333 | 0.333 | Rosendahl [40], Toots [44] |
| **Overall** |  |  |  |  |  |  | **1.0000** | **1.000** | **1.000** |  | **6** |

*Tests whether the inverse theory offers a better solution.

^a^ Independent ambulatory participants (independent with or without walking aid)

**Successful case,** unsuccessful case.

**Appendix 4: GRADE Certainty of evidence**

| **GRADE Certainty assessment** | | | | | | | **№ of patients** | | **Relative Effect (95% CI)** | **Certainty** |
| --- | --- | --- | --- | --- | --- | --- | --- | --- | --- | --- |
| **№ of studies** | **Study design** | **Risk of bias** | **Inconsistency** | **Indirectness** | **Imprecision** | **Other considerations** | **Exercises** | **Usual care** |  |  |
| 8 | Randomised controlled trial | No serious concerns ^a^ | Serious (-1)^b^ | Serious (-1)^c^ | Not serious | None | 365 | 345 | **Rate ratio 0.45** (0.34 to 0.59) | ⨁⨁◯◯ LOW |
| 7 | Randomised controlled trial | No serious concerns ^a^ | Not serious ^d^ | Serious (-1)^c^ | Not serious | Serious (-1)^e^ | 379 | 357 | **Risk ratio 0.66** (0.53 to 0.82) | ⨁⨁◯◯ LOW |

^a^ Effect estimate similar with exclusion of trials at high risk of bias for random sequence generation, allocation concealment, attrition bias, baseline imbalance, method of ascertaining falls

^b^ I^2^ = 60%

^c^ Based on subgroup analysis from QCA items scored, which are not always clearly reported and qualitatively rated

^d^ I^2^ = 0%

^e^ Publication bias strongly suspected based on Funnel plot (see Figure S5 in supplementary materials of Dyer and colleagues [10])
